# Let’s shine a light on fungal infections: A noninvasive imaging toolbox

**DOI:** 10.1371/journal.ppat.1008257

**Published:** 2020-03-05

**Authors:** Katrien Van Dyck, Ona Rogiers, Greetje Vande Velde, Patrick Van Dijck

**Affiliations:** 1 Laboratory of molecular cell biology, Institute of botany and microbiology, Department of biology, KU Leuven, Leuven, Belgium; 2 VIB center for microbiology, Leuven, Belgium; 3 Center for Inflammation Research, VIB, Technologiepark, Zwijnaarde, Belgium; 4 Department of Internal Medicine, Ghent University, Technologiepark, Zwijnaarde, Belgium; 5 Biomedical MRI/ MoSAIC, Dept. Imaging & Pathology, KU Leuven, Leuven, Belgium; University of Maryland, Baltimore, UNITED STATES

## Why is the gold standard not so bright anymore?

The rising prevalence of mycoses over the past 20 years has led to the development of more advanced technologies to study fungal pathogenesis. However, up to date research in laboratory settings still greatly relies on conventional methods such as histological examinations, microbiological cultures, and enumeration of colony forming units. In preclinical settings, these ex vivo approaches lack in providing information on the dynamics of the infection in time and space and do not account for interindividual variation of the disease progression [1, 2]. In addition, these experimental gold standards are endpoint assays where fungal load is analyzed postmortem, resulting in only one time point per animal, thus requiring large groups of animals. Preclinical noninvasive imaging techniques are increasingly used in research because they have the potential to deliver dynamic information on individual animals on a relevant time scale. Moreover, multimodal imaging (i.e., the combination of different imaging techniques and imaging scales) could provide us the complementary information to fully investigate fungal disease progression, host responses, and therapeutic efficacy [3]. This Review highlights the potential of using advanced noninvasive imaging techniques to address biological questions within the field of pathogenic fungi.

## How can we look inside from the outside?

**Fig 1:**
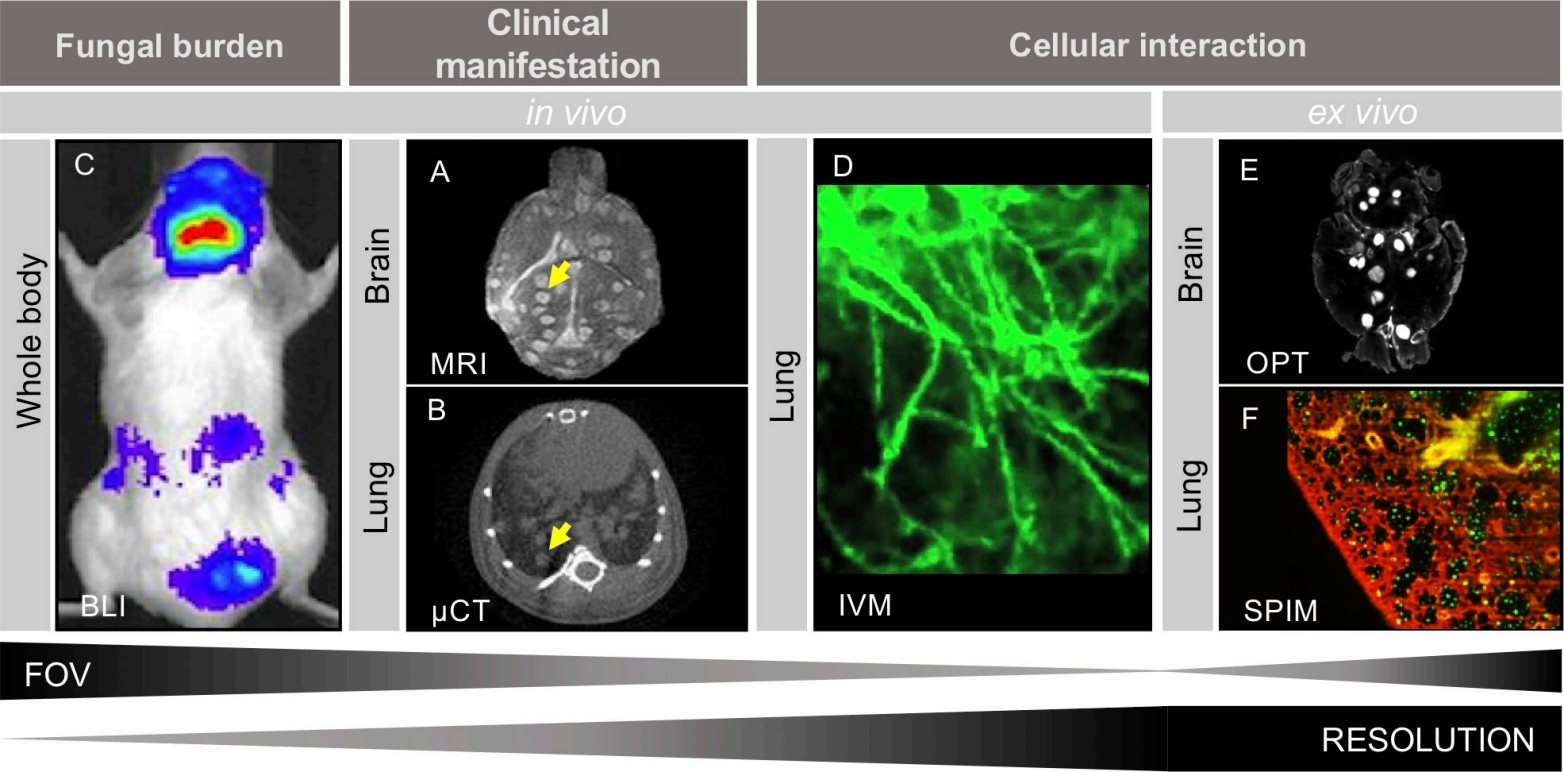
Overview of noninvasive imaging techniques to study fungal infections. A) MR image of the brain from the same *C*. *neoformans* infected mouse on day 5 postinfection. Arrow indicates hyperintense fungal lesions in the brain [2]. B) μCT of pulmonary *C*. *neoformans* infection established upon intranasal inoculation. Deposition of dense tissue lesions in the lungs display fungal load, indicated by the arrow [2]. C) BLI of *C*. *neoformans* tail-vein–infected mouse. Luminescent signal detected from fungal dissemination to brain and abdominal regions on day 7 postinfection [2]. D) IVM image of FITC-labeled *C*. *neoformans* (green) in the mouse brain. An intravenous injection of PE-labeled anti-PECAM-1 antibodies was used to label the brain vasculature (red) [26]. E) IVM image obtained by FCFM through insertion of a probe via the mouth and trachea into the lungs of anesthetized free-breathing mice. GFP-expressing *Aspergillus fumigatus* (green) hyphal structures on lung tissue are visualized [1]. F) OPT image of *C*. *neoformans* infected mouse brain with bright areas representing fluorescently stained cryptococcomas. G) SPIM image of *Cryptococcus gattii* infected mouse lungs, with cryptococci pseudocolored in green, overlaid on the lung tissue (autofluorescent, red). Fig 1A, 1B and 1C, originating from Van Herp and colleagues, [2] are licensed under CC-BY (http://creativecommons.org/licenses/by/4.0/). Fig 1D–1F are obtained by the authors. BLI, bioluminescence imaging; FCFM, fibered confocal fluorescence microscopy; FITC, fluorescein isothiocyanate; FOV, field of view; GFP, green fluorescent protein; IVM, intravital microscopy; MR, magnetic resonance; OPT, optical projection tomography; SPIM, selective plane illumination microscopy; μCT, micro-computed tomography.

Magnetic resonance imaging (MRI) can monitor changes in anatomical structures and physiological characteristics of a tissue, such as blood flow, inflammatory changes, and diffusion, in real time in the same animal. This imaging technique is based on the resonating capacity of a single proton within the hydrogen nucleus. The high abundancy of hydrogen in water and fat make it optimal for medical imaging. An MRI scanner applies a strong magnetic field, causing the alignment of all proton spins. Upon transmission of a radiofrequency current, the magnetic field and proton spins are perturbed, and protons absorb energy from the magnetic field. In turn, a sudden drop in the magnetic field causes the precession of protons, and this process produces a radio signal that is translated into an image. Different tissues can be distinguished by differential precession rates. Using MRI, local inflammation can be evaluated through labeling of phagocytes with ultra-small particle iron–oxide (USPIO) [4]. During infection, recruited phagocytes internalize the administered USPIOs and become magnetic, creating a detectable negative contrast during imaging. Similarly, macrophages can be loaded with a fluorinated contrast agent, creating a positive signal [5]. Combined with proton MRI, this successfully quantified the hyperintense fungal lung lesions within a mouse model of invasive pulmonary aspergillosis [3, 6]. In the context of brain infection, MRI monitors the dissemination and progression of *Cryptococcus neoformans* in infected animals (Fig 1A) [2]. Moreover, MRI can track the loss of blood–brain barrier (BBB) integrity upon administration of an intravascular contrast agent in the metastatic stage of candidiasis [4].

Micro–computed tomography (micro-CT) also allows us to monitor in vivo changes from the outside during pathogenesis. This imaging technique is based on X-ray transmission of a beam of accelerated electrons that is emitted and travels through the animal. The detector measures and converts the radiation, attenuated from passing through the sample, into a 2D digital image. The X-ray source and detector rotate around the animal, continuously creating radiographs at different positions that are afterwards reconstructed into a 3D representation. Based on the inherent density differences and therefore excellent contrast between tissue and air in the lungs, micro-CT can be used to visually and quantitatively evaluate pulmonary infections. Several biomarkers can be derived that quantify different aspects in mouse models of lung disease, i.e., lesion volume, which reflects the fungal burden; total lung volume, which reflects host response to compensate for the loss of air spaces due to infection lesions; and aerated lung volume, which corresponds to a functional read-out [6]. In models of pulmonary cryptococcosis or aspergillosis, growing fungal lesions within the lungs of infected animals were monitored and quantified using micro-CT. Cryptococcosis lung lesions appeared as gray patches in contrast to the black background corresponding to the aerates part of the lung (Fig 1B) [2, 6].

Although both MRI and micro-CT visualize detailed in vivo changes within infected organs of interest during fungal infection, a different approach is required to estimate the fungal burden directly and zoom out to consider dissemination within a host.

## Can we get the bigger picture?

Over the years, several robust fungal bioreporters have been constructed that enable direct and repeated visualization of fungal cells and their distribution dynamically in a living host. To track the most common opportunistic fungal infections (candidiasis and aspergillosis), the natural phenomenon of bioluminescence was successfully adapted to generate genetically modified *Candida albicans*, *Candida glabrata*, and *A*. *fumigatus* that stably express a luciferase reporter gene, which in turn generates photons upon catalyzing the oxidation of its respective substrate [7]. The emitted photon flux from the fungal reporter cells is externally detected by sensitive photon detectors and thus offers the possibility to follow fungal cell proliferation in real time and noninvasively throughout the whole body of the host. Continuous advancements in bioluminescence imaging (BLI) reporter systems have resulted in the successful adoption of two luciferases (*Gaussia* and firefly luciferase) for different fungal pathogens, such as *C*. *neoformans*, *C*. *glabrata*, and *A*. *terreus* [2, 8, 9]. Sensitive BLI visualized late-stage dissemination to the brain after primary infection of the lung with *C*. *neoformans* (Fig 1C) [2]. The latest optimization in *C*. *albicans* research is the construction of the red-shifted firefly luciferase [10]. The field of use of this reporter system has expanded to monitoring cutaneous, subcutaneous, vaginal, oropharyngeal, deep-seated, and biofilm infections [11–17]. In addition, BLI has increased the in vivo screening possibilities to monitor antifungal drug efficacy during infection [9, 15, 18]. Although significant progress was made in recent years, the available BLI systems encounter several limitations that will certainly remain challenges in the field. The need to administer a substrate and the oxygen dependency of the luciferase makes that the quantification accuracy depends on bio-availability, distribution, and the presence of oxygen into infected sites. Along with the development of bioluminescent reporters, fluorescent in vivo imaging made advancements as a substrate-free alternative. Fluorescent proteins, such as GFP and DsRed, are easily expressed in fungal cells. Modifications to excitation and emission wavelengths solved challenges such as faint brightness or background noise [19]. The best results for in vivo imaging of *C*. *albicans* were achieved with the new near-infrared spectrum proteins (iRFPs) [20]. Optical reporters supplement the existing palette of tools and permit researchers to evaluate infection progression and therapy outcome in real time, visualizing the whole-body effect on dissemination within individual animals.

## Are there more pieces to this puzzle?

Combining anatomical and optical whole-body imaging techniques allows us to monitor the fungal burden and clinical manifestations in individual animals over time. To zoom in from whole-body and organ-scale information, modern technologies are under development to provide us the opportunity to visualize cellular processes with a high resolution. Intravital microscopy (IVM) enables us to image pathogens in a complex biological environment, such as living tissues and organs [1]. Two-photon intravital imaging (2P-IVM) is used for fluorescence imaging of tissues at microscopic resolution [21] through obtaining access to sites of interest by implantation of an optical window. This technique is often used to study the migration of fungal pathogens across the BBB (Fig 1D) [22]. However, 2P-IVM is technically challenging and limited to superficial interrogation of tissues. To gain access to deeper infection sites, such as the lungs, IVM is applied by using a fibered confocal fluorescence microscopy (FCFM) approach instead. With such a technique, infections can be monitored in a minimally invasive way as the flexible optical probe is inserted in the body and placed directly at the site of interest [1, 22]. In a preclinical context, fluorescently labeled micro-organisms could be used for detailed investigations of interactions with the host at a cellular level. Recently, an IVM by FCFM procedure was optimized to repeatedly image *A*. *fumigatus* and *C*. *gattii* infections in mouse lungs in a minimally invasive way (Fig 1E) [1]. Currently, IVM by FCFM is limited to visualize only two colors, whereas 2P-IVM can be applied with multiple exogenous fluorescent markers [21].

## Did we overlook a missing link?

A technological gap still exists between whole-body imaging, which provides a large field of view (FOV) but lacks cellular resolution, and microscopic techniques, which offer this cellular resolution but only for a very limited FOV. Mesoscopic imaging techniques may provide us this missing link by looking at cellular details of intact biological systems, such as whole organs [23, 24]. Optical projection tomography (OPT) can produce high resolution 3D images by recording a series of optical projections while the optically cleared sample is rotated. Since light penetration through tissue is limited, the sample is first rendered transparent by optical clearing. Further zooming-in at even higher resolution is possible with selective plane illumination microscopy (SPIM), a fluorescence light sheet microscopy technique that uses a thin sheet of laser light to optically section the fluorophore-labeled samples. A stack of images is acquired to 3D-image an intact whole-organ sample. Moreover, as OPT and SPIM offer the possibility to multiplex different fluorescent signals, it can be developed to look both at labeled pathogens and host cells. OPT and SPIM are successfully used in embryology and diabetes research [24, 25] and are currently being optimized for fungal infections as well (Fig 1F and 1G).

To conclude, a multimodal imaging approach is crucial to overcome the limitations of the individual techniques and to obtain all pieces of the puzzle. Although the scientific benefits of these techniques are obvious, additional factors to consider are the relatively high investment cost for equipment, availability of the appropriate infrastructure, and requirement of a biosafety level 2 facility for the implementation for fungal research. In fact, major advancements and insights have been uncovered by applying these techniques in simplified animal models of fungal infections. Future challenges comprise the imaging of pathogenic fungi in more complex models to provide additional insights on host–pathogen or pathogen–pathogen interactions. This entails imaging of differentially labeled cells, such as host versus fungal cells or mixed pathogenic species, considering the polymicrobial nature of the host.
